# KanuriSenti: A novel dataset for sentiment analysis in the under-resourced Kanuri language

**DOI:** 10.1016/j.dib.2025.111758

**Published:** 2025-06-07

**Authors:** Bashir Maina Saleh, Saurabh Bilgaiyan, Santwana Sagnika

**Affiliations:** School of Computer Engineering, KIIT, Deemed to be University, Bhubaneswar, Odisha, 751024, India

**Keywords:** Kanuri dataset, NLP, Sentiment analysis, Opinion mining, Under-resourced language, Low-resource Language

## Abstract

This paper presents **KanuriSenti**, a novel sentiment analysis dataset developed for **Kanuri**, a major yet under-resourced language spoken across Nigeria and the Lake Chad region. The dataset addresses a critical gap in Natural Language Processing by providing structured and sentiment-annotated Kanuri data, which has been largely absent from existing resources. KanuriSenti consists of a lexicon-based dataset containing over 10,000 entries labeled with sentiment polarity (positive, negative, neutral), and an affective E-ANEW-style dataset annotated across Valence, Arousal, and Dominance dimensions by native Kanuri speakers. Annotation consistency and reliability was validated using Cohen’s Kappa, and lexical richness was measured using the Type-Token Ratio, confirming our dataset's suitability for sentiment-related tasks. While the dataset is designed specifically for **sentiment analysis**, its cultural and linguistic authenticity makes it a valuable benchmark for evaluating sentiment models in low-resource language settings and advancing equitable language technology in Africa.

Specifications Table

**Table:** 

Subject	Computer Sciences
Specific subject area	Sentiment Analysis Dataset for Under-Resourced Languages in Natural Language Processing
Type of data	Table, Raw.
Data collection	We first leveraged the PanLex lexical database and a Manga Kanuri Dictionary to compile an initial Kanuri wordlist as a foundation for our dataset. We then partnered with native Kanuri speakers from Yobe State University, the University of Maiduguri, and surrounding communities to validate and expand these entries, gather short texts, and ultimately build a comprehensive annotated corpus. Thereafter some frequently occurring emotional words were derived and emotional rating was carried out for the valence, arousal and dominance of those words as e-anew data.
Data source location	The data were collected in Yobe State University and University of Maiduguri, in Northeast Nigeria. The wordlist are gotten from: https://vocab.panlex.org/knc-000 [15] A Dictionary of Manga, a Kanuri Language of Eastern Niger and NE Nigeria [14] Nigeria: Yobe State University Damaturu, University of Maiduguri.
Data accessibility	Repository name: KanuriSenti Dataset Data identification number: 10.17632/pcfvh7r6nd.1 Direct URL to data: https://data.mendeley.com/datasets/pcfvh7r6nd/1
Related research article	None

## 1 Value of the Data

• This dataset addresses a critical research gap by offering a much-needed resource for sentiment analysis in under-resourced Kanuri language. By providing a carefully curated sentiment analysis corpus, it enables deeper research and technological advancements for the language overlooked in NLP research.
• Derived from real-world expressions, the dataset serves as a robust benchmark for researchers looking to test and refine multilingual or low-resource language modeling techniques.
• By capturing culturally and contextually nuanced sentiments, it enables more accurate model training and evaluation, while the involvement of native Kanuri speakers ensures authenticity and fosters community-driven data validation.
• Beyond sentiment analysis, the dataset can be repurposed for diverse NLP tasks such as part-of-speech tagging, morphology, and machine translation, thereby broadening opportunities for comprehensive Kanuri language NLP research.

## 2 Background

Natural Language Processing (NLP) is a field of artificial intelligence that enables machines to understand and interpret human language [[1], [2], [3]]. A key application of NLP is sentiment analysis, which extracts opinions and emotions from text [4]. However, NLP faces significant challenges in low-resource languages [5], such as Kanuri, spoken primarily in Nigeria, Niger, Chad, and Cameroon [6].

Unlike high-resource languages with abundant NLP resources [5], Kanuri lacks essential corpora, annotated datasets, and tools required for developing sentiment analysis models [5]. This made it difficult for Sentiment Analysis and other NLP researches in Kanuri.

Despite several studies that have developed sentiment analysis datasets for other low-resource languages such as Bangla [7], Sesotho [8], Hausa, Igbo, Nigerian Pidgin, and Yoruba [9], the Kanuri remains largely overlooked.

To address the critical lack of structured linguistic resources for Kanuri Language, we compiled a sentiment-annotated dataset that directly responds to this gap. The absence of such resources has long hindered the development of technological applications for Kanuri. This paper presents the first publicly available sentiment analysis lexicon- based and E-anew dataset for the Kanuri language, aiming to bridge this research gap. The dataset is openly accessible via the Mendeley Data repository [10]. The dataset prioritizes native speaker input to ensure lexical and contextual fidelity.

## 3 Data Description

The dataset comprises of two Excel (.xlsx) files, each represented in tabular format with detailed descriptions. The first file named “KanuriSentiUpdatedDateset.xlsx” presents the first Lexicon-based dataset comprising of 10,356 records with three columns namely Kanuri, English translation and Polarity(which contains either positive, negative or neutral). This is suited for classification tasks, such as training a model (e.g. logistic regression or transformer) to assign one of the three sentiment labels to Kanuri text.

The second file named “E-Anew_Dataset.xlsx” is an e-anew dataset comprising of 65 commonly used words for sentiments in Kanuri with their English translation and score for the Valence, Arousal and Dominance. These scores are average from three different annotators/assessors of the words for the VAD, their individual scores and how the average came to be is also attached in the third file named “E-anew_with_annotators.xlsx” in the repository. This VAD dataset is suited for regression tasks, enabling models to predict fine-grained emotion intensity along each dimension (e.g. via linear regression or neural networks) (Table 1).

**Table 1:** Overview of the Lexicon-based dataset.

S/N	Polarity	Number of Entries
1	Positive	2886
2	Neutral	4499
3	Negative	2971
TOTAL		10,356

It is worthy of note that the datasets are provided in Excel (.xlsx) format, for those who prefer working with “.csv”, the files can be easily converted using Microsoft Excel, Python (via Pandas), LibreOffice, or Google Sheets. We recommend using UTF-8 encoding when saving the files to preserve Kanuri characters (Table 2).

**Table 2:** A portion of the Lexicon-based dataset.

S/N	Kanuri Text	Polarity	English Meaning
1	suro ajiyen shiro ngəlawo	Positive	best in class
2	rokkonzən cida diwodə kəji	Positive	a pleasure to work with you
3	wande tadaro wande rinəmi	Neutral	do not be afraid of a child
4	awo dai an diya yak sina do tawatti kiye	Negative	i am not sure what it is that makes me estranged
5	sai kafinta karunzo bannato lai ziya	Negative	the carpenter got angry and left

To ensure the reliability of the annotations, Cohen’s Kappa in equation 1—a statistical measure commonly used to evaluate inter-annotator agreement—was employed. Cohen’s Kappa quantifies the extent to which the observed agreement among annotators surpasses chance-level expectations [11]. Given the inherent subjectivity in emotional ratings, calculating Cohen’s Kappa validated the consistency and objectivity of annotators' judgments across the Valence, Arousal, and Dominance dimensions, thus confirming the credibility and quality of the annotated data (Table 3).

(1) $$\kappa =\frac{\text{Po}-\text{Pe}}{1-\text{Pe}}$$

(2) $$Where\;\text{Po}=\frac{\text{Number}\;\text{of}\;\text{agreements}}{\text{Total}\;\text{number}\;\text{of}\;\text{ratings}}$$

(3) $$\text{Pe}=\sum_{i=0}^{k}P_{i1}\times P_{i2}$$

**Table 3:** A portion of the E-anew dataset

S/N	Kanuri	English Translation	Valence	Arousal	Dominance
1	rawoo wa dunoma	Exciting	0.9	0.9	0.7
2	rawoo	hopeful	0.8	0.6	0.5
3	watunoo	disgusting	−0.9	0.8	0.6
4	silwai	vulnerable	−0.7	0.6	0.5
5	karuzaa	Grief	−0.9	0.8	0.4

Also, pi1 and pi2 represent the *proportions* (or probabilities) that *annotator 1* and *annotator 2*, respectively, assign *a particular category i.*

Applying this formula on the e-anew dataset, it can be clarly seen in Table 4 that the Cohen’s Kappa scores indicate moderate to substantial agreement among annotators across Valence, Arousal, and Dominance dimensions, confirming the reliability and consistency of the sentiment annotations in the KanuriSenti dataset. Specifically, the average Kappa values were 0.57 for Valence, 0.44 for Arousal, and 0.61 for Dominance, reflecting the highest agreement in Dominance and the lowest in Arousal. These results affirm that the dataset is dependable for sentiment analysis tasks, especially in evaluating affective dimensions in low-resource language contexts.

**Table 4:** Inter-annotator agreement measured by Cohen’s Kappa across valence, arousal, and dominance.

Emotional Dimension	Annotator 1 vs 2	Annotator 1 vs 3	Annotator 2 vs 3	Average Kappa
Valence	0.507109	0.569536	0.632075	0.569574
Arousal	0.466922	0.433469	0.424779	0.441723
Dominance	0.564593	0.629981	0.630332	0.608302

Also, we checked the Lexical richness in the dataset using the Type-Token Ratio (TTR) formula given by [12] below:

(4) $$TTR=\frac{Total\;No\;of\;words}{No\;of\;Unique\;words}$$

As seen in Table 5, the range of the lexical richness is mostly ≈0.35 which indicates the dataset is healthy and typical for general-purpose sentiment datasets. It indicates our dataset has a reasonably diverse vocabulary, suitable for robust sentiment analysis as suggested by [13].

**Table 5:** Lexical richness of polarity.

S/N	Polarity		Kanuri_Text
1	Negative	Total_Words	8464
		Unique_Words	2901
		Lexical_Richness	0.3427
2	Neutral	Total_Words	11388
		Unique_Words	4023
		Lexical_Richness	0.3533
3	Positive	Total_Words	7878
		Unique_Words	2717
		Lexical_Richness	0.3449

In preparing KanuriSenti for release, we applied the following data‐cleaning steps to every entry:

I. Lowercasing: All Kanuri and English text were converted to lowercase to reduce sparsity during feature extraction.
II. Manual spelling correction: A panel of three native Kanuri speakers reviewed the corpus and standardized frequent orthographic variants (for example, “karuzâ” was corrected to “karuzaa”).
III. Token filtering: Entries longer than three words or containing any non-alphabetic characters were removed to ensure consistency of granularity and content.
IV. Missing-value handling: Any record lacking either its translation or its sentiment/polarity or VAD annotation was excluded from the final dataset.

These steps guarantee that KanuriSenti is both high-quality and immediately suitable for downstream NLP tasks.

**Fig. 1:**
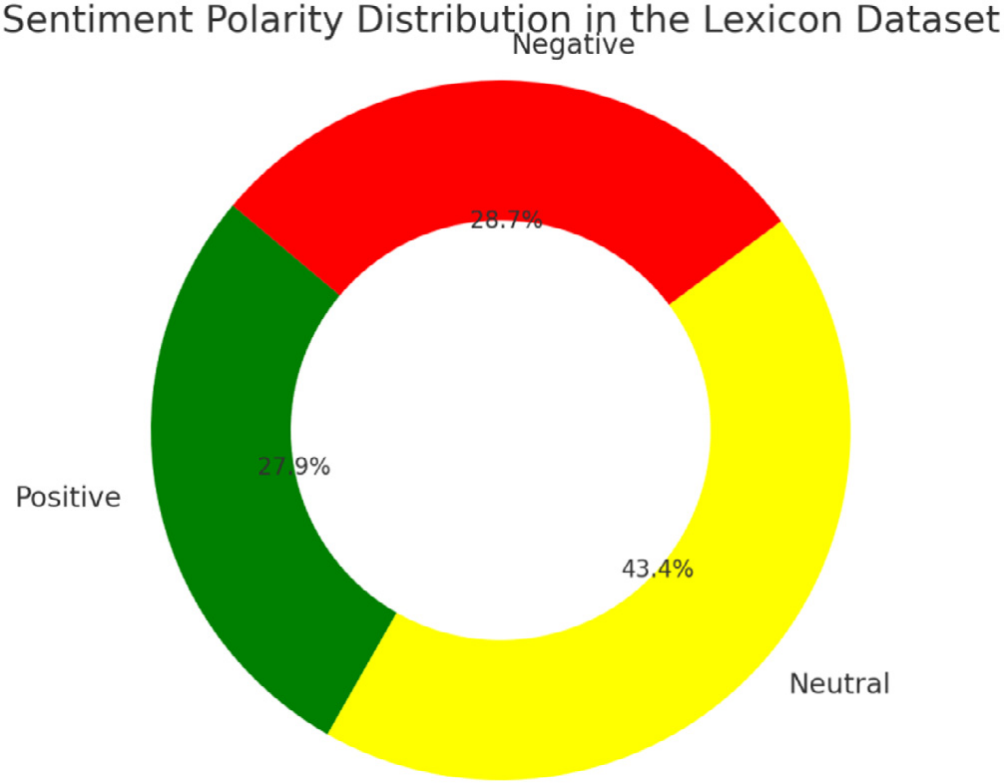
Polarity distribution in the lexicon dataset.

As illustrated by the donut chart in Fig. 1, our dataset exhibits a mild class imbalance: neutral examples comprise 43.4 % of the corpus, while positive and negative account for 27.9 % and 28.7 %, respectively. Although both polarities are nearly equally represented, the predominance of neutral instances suggests that downstream classifiers should employ stratified sampling or apply class‐weighting (or oversampling/undersampling) strategies to mitigate potential bias during training.

### 3.1 Simple baseline

We employed both a rule‐based lexicon method and a supervised statistical baseline to ensure immediate, zero‐code usability while establishing a clear performance benchmark for future usage and enhancements.

### 3.2 Rule‐based usage example

Here, we illustrate our lexicon lookups on three Kanuri entries in Table 6, where for each entry, we extract its polarity label and VAD scores, then compute:

• Polarity classification by taking the lexicon’s polarity value. For example, the first entry “karu kəji” the lexicon value gives positive, so the entry is classified as positive.
• Emotion regression by averaging its VAD scores. For example, the entry “karu kəji”: $Avg\;VAD=\frac{0.90+0.60+0.50}{3}$ ≈0.67 .

**Table 6:** Rule‐based usage example.

ID	Kanuri	English	Polarity	Valence	Arousal	Dominance
1	karu kəji	Happy	Positive	0.90	0.60	0.50
2	karuza	Sad	Negative	–0.80	0.60	0.40
3	grata	Angry	Negative	–0.90	0.90	0.80

### 3.3 Statistical baseline classifier

We also provide a reproducible supervised baseline Using TF–IDF features and a logistic-regression model (80/20 stratified split), our classifier achieves Accuracy 0.6704 and Macro-F1 0.6575. Complete implementation details, hyperparameters, and evaluation scripts are available in our GitHub repository .

This simple dual‐baseline approach balances ease of use with rigorous benchmarking, laying a robust foundation for all future Kanuri sentiment analysis research.

## 4 Experimental Design, Materials and Methods

After leveraging the PanLex lexical database [15] and Manga Kanuri dictionary [14] to compile an initial Kanuri wordlist as a foundation for our dataset, we then partnered with native Kanuri speakers from Yobe State University, the University of Maiduguri, and surrounding communities to validate and expand these entries, gather short texts, and ultimately build a comprehensive annotated corpus. Throughout this process, we prioritized native speaker input to ensure lexical and contextual fidelity. Translations and annotations were done by these bilingual Kanuri speakers referred to as AN1, AN2,AN3,AN4 and AN5 in Fig. 1.

**Fig. 2:**
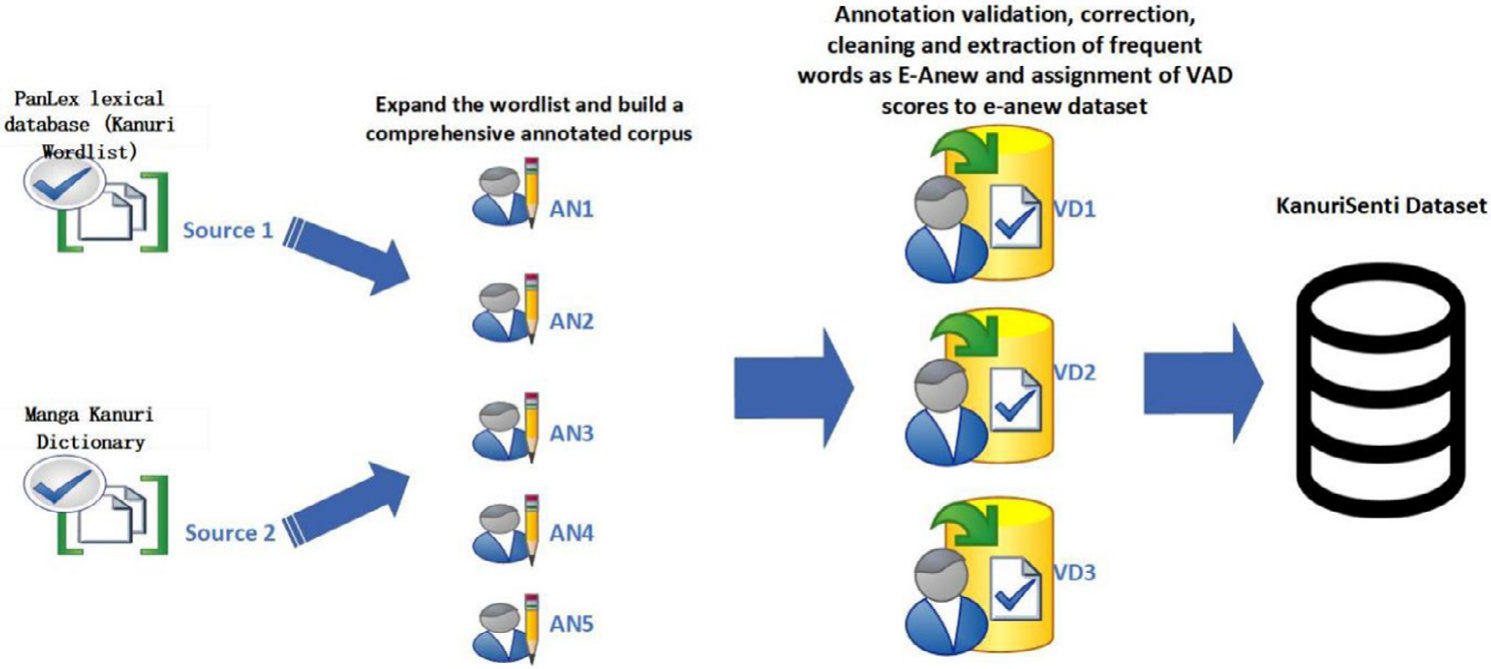
Dataset curation process.

Another set of annotators referred to as validators (VD1, VD2 and VD3) are then trained to clearly understand the psychological dimensions they evaluate, which include Valence (how pleasant or unpleasant a word is), Arousal (the intensity or level of emotional activation elicited by the word), and Dominance (the degree of control or power associated with the emotional response). They utilize the Self-Assessment Manikin (SAM) as their measurement instrument, a pictorial scale designed to assist in objectively assessing these emotional dimensions (Fig. 2).

During annotation, the validators follow a structured process wherein frequently occurring words from lexicon-based curated datasets presented to them via Google Sheets were compiled during the validation phase. They evaluate their immediate emotional responses to each word and rate each psychological dimension separately giving their respective scores to the words.

## Limitations

A key limitation of the E-ANEW dataset is its limited coverage of affective vocabulary, which may constrain its ability to generalize across diverse emotional expressions.

## Ethics Statement

We have read and follow the ethical requirements for publication in Data in Brief and confirm that the current work does not involve human subjects, animal experiments, or any data collected from social media platforms. Furthermore, the data obtained from the two sources—PanLex and the Kanuri Manga Dictionary—are publicly available and not subject to any restrictions or licensing requirements for copying, editing, or reuse.

## CRediT Author Statement

**Bashir Maina Saleh:** Conceptualization, Data curation, Writing - original draft; **Saurabh Bilgaiyan:** Supervision and Reviewing; **Santwana Sagnika:** Supervision, Writing - review, and Validation.

## Data Availability

Mendeley DataKanuriSenti Dataset (Original data).
